# Central venous oxygen saturation and blood lactate levels during cardiopulmonary bypass are associated with outcome after pediatric cardiac surgery

**DOI:** 10.1186/cc9217

**Published:** 2010-08-04

**Authors:** Marco Ranucci, Giuseppe Isgrò, Concetta Carlucci, Teresa De La Torre, Stefania Enginoli, Alessandro Frigiola

**Affiliations:** 1 Department of Cardiothoracic and Vascular Anesthesia and ICU, IRCCS Policlinico San Donato, Via Morandi 30, 20097 San Donato Milanese (Milan), Italy; 2 Department of Cardiac Surgery, IRCCS Policlinico San Donato, Via Morandi 30, 20097 San Donato Milanese (Milan), Italy

## Abstract

**Introduction:**

Central venous oxygen saturation and blood lactate are different indices of the adequacy of oxygen delivery to the oxygen needs. In pediatric cardiac surgery, lactate level and kinetics during and after cardiopulmonary bypass are associated with outcome variables. The aim of this study was to explore the hypothesis that the lowest central venous oxygen saturation and the peak lactate value during cardiopulmonary bypass, used alone or in combination, may be predictive of major morbidity and mortality in pediatric cardiac surgery.

**Methods:**

We conducted a retrospective analysis of 256 pediatric (younger than 6 years) patients who had undergone cardiac surgery with continuous monitoring of central venous oxygen saturation and serial measurement of blood lactate.

**Results:**

Peak lactate was significantly increased when the nadir central venous oxygen saturation was < 68%. Both nadir central venous oxygen saturation and peak lactate during cardiopulmonary bypass were independently associated with major morbidity and mortality, with the same accuracy for major morbidity and a higher accuracy of peak lactate for mortality. A combined index (central venous oxygen saturation < 68% and peak lactate > 3 mmol/L) provided the highest sensitivity and specificity for major morbidity, with a positive predictive value of 89%.

**Conclusions:**

The combination of a continuous monitoring of central venous oxygen saturation and serial measurements of blood lactate during cardiopulmonary bypass may offer a predictive index for major morbidity after cardiac operations in pediatric patients. This study generates the hypothesis that strategies aimed to preserve oxygen delivery during cardiopulmonary bypass may reduce the occurrence of low values of central venous oxygen saturation and elevated lactate levels. Further studies should consider this hypothesis and take into account other time-related factors, such as time of exposure to low values of central venous oxygen saturation and kinetics of lactate formation.

## Introduction

Central (ScVO_2_) and mixed venous oxygen saturation monitoring has a well-defined role for guiding hemodynamic management in adults and children undergoing major surgical operations [1,2]. Its role in critically ill patients has been defined [3,4]. In pediatric cardiac surgery, perioperative goal-directed therapy with continuous ScVO_2 _monitoring is associated with excellent early survival and a low incidence of organ failure after stage 1 palliation for hypoplastic left heart syndrome [5,6].

In cardiac operations, high values of blood lactate have been associated with bad outcomes if detected both during cardiopulmonary bypass (CPB) [7,8] and at the arrival in the intensive care unit (ICU) in adult patients [9]. In pediatric patients undergoing cardiac surgery for congenital heart disease, many studies highlighted the potential role of hyperlactatemia on admission to the ICU as a marker for adverse outcome [10-14], and one study linked hyperlactatemia during CPB with postoperative morbidity and mortality [15]. Studies simultaneously addressing both ScVO_2 _and blood lactates during CPB as potential early predictors of morbidity and mortality in pediatric cardiac operations are still lacking.

At present, venous oxygen saturation may be continuously measured during CPB, by using specific detectors placed in the venous line of the circuit, or by using central venous catheters (CVCs) that incorporate fiberoptic technology for oxygen-saturation measurement. The present study investigates the hypothesis that simultaneous measurement of continuous ScVO_2 _coupled with serial blood lactate determination may provide one or more early markers for postoperative adverse outcomes in pediatric cardiac surgery.

## Materials and methods

This is a retrospective study, approved by our Local Ethics Committee, which waived the need for obtaining written informed consent. All data were retrieved by using our Institutional Database, which includes all the perioperative details and outcome data of our patients; ScVO_2 _and lactate values were retrieved by retrospectively analyzing the perfusion files.

### Study period and patient selection

Continuous ScVO_2 _monitoring for pediatric patients was introduced in our Department in 2007. Therefore, all the pediatric (younger than 18 years) patients undergoing a cardiac operation in the period from January 2007 through October 2009 were considered for being included in this study. This group comprised 732 patients. One hundred thirty-four patients were excluded because they were operated on without CPB. Continuous ScVO_2 _monitoring is usually applied in operations of medium to high complexity; therefore, 254 patients were excluded because of the simple nature of the operation. The remaining 344 patients were analyzed, and a group of 68 patients was excluded because they did not receive continuous SCVO_2 _monitoring.

From the remaining group of 266 patients, 10 patients were excluded because they demonstrated a pre-CPB lactate value higher than 3.0 mmol/L. A final group of 256 nonconsecutive patients was therefore retrieved, and constituted the patient group for this study.

### Data collection

The following data were collected from the Institutional Database or direct analysis of the perfusion files: demographics: age (months), weight (kg), gender; type of surgical operation with Aristotle complexity score [16]; preoperative laboratory data: hematocrit (percentage), platelet count (cells/microliter), prothrombin time (seconds), activated partial thromboplastin time (seconds), antithrombin (percentage), serum creatinine value (milligrams per deciliter); CPB data: CPB duration (minutes), lowest temperature on CPB (degrees Centigrade), use of blood prime, ScVO_2 _values (percentage), and lactate values (mmol/L). Lactate values were obtained from standard arterial blood gas analysis (Nova Biomedical, Waltham, MA).

ScVO_2 _values are routinely recorded in the perfusion files at an interval of 10 minutes, whereas lactate values are recorded in correspondence with the arterial blood gas analysis, at intervals of 20 to 30 minutes. In our daily practice, the perfusionist is instructed not to record low values of ScVO_2 _maintained for a short period of time (< 5 minutes) because of surgical maneuvers and the need for decreasing pump flow according to the surgeon's instructions. Therefore, the ScVO_2 _values recorded are usually maintained for a time of at least 10 minutes, until the subsequent recording.

For each patient, we detected the *nadir ScVO_2 _*value (lowest SCVO_2 _on CPB) and the *peak lactate *value (highest lactate value on CPB).

### ScVO_2 _monitoring details

ScVO_2 _was measured by using a double-lumen CVC inserted through the right internal jugular vein into the superior vena cava, in a position proximal to the insertion of the venous cannulation for CPB. The CVC catheter incorporates fiberoptic technology for oxygen saturation and was released a few years ago for use in neonates and pediatric patients (Pediasat; Edwards Lifesciences, Irvine, CA). Details of the positioning were previously published by our group, as well as validation data [17]. In particular, our protocol avoids entering the right atrium in all the procedures requiring the opening of this chamber, to obtain hemoglobin saturation data even during CPB. ScVO_2 _data are obtained by connecting the Pediasat CVC to a dedicated monitor (Vigileo; Edwards Lifesciences, Irvine, CA).

### Anesthesia, cardiopulmonary bypass, and cardiac surgery technique

Anesthesia was carried out according to our institutional practice. Induction of anesthesia was achieved with intravenous midazolam. A high-dose opioid anesthetic (fentanyl, 50 μg/kg) was used for maintenance of anesthesia and supplemented with midazolam and sevoflurane as tolerated. Neuromuscular blockade was achieved with vecuronium or atracurium. All patients underwent endotracheal intubation and were mechanically ventilated. Standard monitoring was used, which included a radial or femoral artery catheter for measurement of systemic arterial blood pressure and intermittent blood sampling, a double-lumen right internal jugular catheter, and esophageal and rectal temperature probes.

Cardiac cannulation was performed after intravenous administration of 300 IU/kg of unfractionated heparin and only after an activated clotting time of longer than 450 seconds was achieved. Additional heparin boluses were used to maintain an activated clotting time in this range before and during CPB. Double venous cannulation of the superior and inferior vena cava was generally performed. The arterial cannula was placed into the ascending aorta. The CPB circuit included a hollow fiber oxygenator (Dideco D901 or D902; Sorin Group, Mirandola, Italy) with an arterial line filter and a centrifugal pump (Bio-Medicus; Medtronic, Minneapolis, MN). In the blood-primed patients, the CPB circuit was primed with a solution containing red blood cells (RBCs) and a 4% albumin solution. The solution was titrated to reach a hematocrit value of 30% once the patient was connected to the circuit and CPB was initiated. The total priming volume varied between 350 mL and 450 mL. Therefore, the amount of RBCs used in the priming solution varied according to the patient's baseline hematocrit, weight, and the priming volume used. In all patients, less than a 250-mL volume of RBCs and only one bag of stored RBCs were used for priming the circuit. Non-blood-primed patients received a 4% albumin solution for priming the CPB circuit. CPB flow was targeted at 150 mL/kg and subsequently adjusted according to the patient's temperature.

The target patient temperature was chosen by the surgeon based on the type or surgical procedure being performed and personal preferences. All procedures were performed by using a regimen of mild (32°C to 34°C), moderate (26°C to 31°C), or deep (20°C to 25°C) hypothermia. Patients were treated with an alpha-stat strategy if mild hypothermia was used and with a pH-stat strategy if moderate or deep hypothermia was used.

Cardiac arrest was obtained and maintained by using antegrade intermittent blood cardioplegia. After completion of the CPB and removal of the cannulas, heparin was reversed by using protamine sulfate at a 1:1 ratio.

### Outcome data

The following outcome data were recorded: mechanical ventilation time (hours); ICU stay (days); neurologic complications (stroke, choreoathetosis, seizures); acute renal failure (need for renal-replacement therapy); pulmonary complications (respiratory distress syndrome; poor gas exchange resulting in a delayed weaning from mechanical ventilation; pneumonia); gastroenteric complications (necrotizing enterocholitis, mesenteric ischemia, gastric bleeding); need for extracorporeal membrane oxygenation or ventricular-assist device; or sepsis (with positive blood cultures). Major morbidity was defined as the presence of at least one of these complications, with or without hospital mortality. Hospital mortality was defined as mortality occurring during the hospital stay.

### Statistics

Continuous variables were explored for normality of distribution by using a Kolmogorov-Smirnov test, and in case of nonnormal distribution were presented as median and interquartile range and analyzed with nonparametric tests. Categoric data are presented as number and percentage. The Kruskal-Wallis test was applied for comparing between-group differences. Correlation between continuous variables was assessed by using a linear or polynomial regression analysis, producing an r^2 ^correlation coefficient.

Association of independent variables with the two outcome measurements (major morbidity and mortality) was explored by using a logistic regression analysis. To control for other covariates, multivariate logistic regression analysis was used, producing odds ratios with a 95% confidence interval.

The predictive accuracy of nadir ScVO_2 _and peak lactate for major morbidity and mortality was explored by using the receiver operating characteristic (ROC) curve and the relative area under the curve (AUC). For each parameter, different cut-off points were tested for sensitivity, specificity, and positive and negative predictive power.

A *P *value < 0.05 was considered to be significant for all statistical tests. Statistical calculations were performed by using a computerized statistical program (SPSS 13.0; Chicago, IL).

## Results

For the 256 patients studied, operation details are shown in Table 1. The group "miscellaneous" comprises a number of different operations, including total venous anomalous pulmonary return, valve repairs, double-outlet right ventricle, conduits replacement, and pulmonary artery reconstruction. The higher major morbidity and mortality rate was reached in Norwood operation, followed by miscellaneous operations and arterial switch operation. Major morbidity was observed in 27 (10.5%) patients. Neurologic complications were observed in three (1.2%) patients, acute renal failure in six (2.3%) patients, pulmonary complications in 15 (5.9%) patients, gastroenteric complications in two (0.8%) patients, and sepsis in 10 (3.9%) patients. Ventricular-assist devices were used in three (1.2%) patients. Ten patients (3.9%) did not survive.

**Table 1:** Surgical description with major morbidity and mortality rates

Operation		Major morbidity		Mortality	
			
	Number	Number	%	Number	%
Ventricular septal defect	90	3	3.3	0	0
Tetralogy of Fallot	41	4	9.8	2	4.9
Complete atrioventricular canal	36	4	11.1	1	2.8
Arterial switch operation	27	8	29.6	2	7.4
Cavo-pulmonary connection	7	0	0	0	0
Truncus arteriosus	6	1	16.7	0	0
Norwood operation	3	1	33.3	1	33.3
Miscellaneous	46	6	13	4	8.7
**Total**	**256**	**27**	**10.5**	**10**	**3.9**

Table 2 reports the demographics, and the preoperative and operative details of the population. Patients with postoperative major morbidity or mortality had a higher-risk profile, characterized by a significantly younger age, smaller weight, higher Aristotle score, and higher serum creatinine level. Preoperative hematocrit was significantly higher in patients with major morbidity or mortality, indicating a higher rate of cyanotic patients in these groups.

**Table 2:** Demographics and intraoperative details between patients without major morbidity, patients with major morbidity, and nonsurvivors

Factor	No major morbidity (*n *= 228)	Major morbidity (*n *= 27)	Nonsurvivors (*n *= 10)	*P *value^a^	*P *value^b^
Age (months)	8 (4-12)	5 (0.7-9.5)	1 (0.4-5)	0.005	0.004
Weight (kg)	6.6 (4.9-8)	4.2 (3-6.6)	3.3 (2.6-5.9)	0.001	0.003
Aristotle score	7.5 (6-8)	8 (7.5-11)	8 (7.5-11)	0.001	0.01
Hematocrit (%)	34 (31-37)	36 (33-38.5)	38 (35.5-42)	0.013	0.001
Serum creatinine (mg/dL)	0.3 (0.2-0.4)	0.4 (0.3-0.5)	0.6 (0.3-0.9)	0.031	0.002
CPB duration (min)	78 (56-106)	130 (81-204)	138 (112-286)	0.001	0.001
Lowest temperature (°C)	30 (28-31)	28 (27-30)	27 (25-29)	0.001	0.001
Nadir ScVO2 (%)	74 (72-77)	68 (59-74)	67 (52-76)	0.001	0.009
Peak lactate (mmol/L)	1.8 (1.5-2.4)	2.9 (1.9-4)	4 (2.7-8.8)	0.001	0.001

Data are expressed as median (interquartile range). Comparison of groups by Kruskal-Wallis test. ^a^Major morbidity versus no major morbidity. ^b^Nonsurvivors vs. no major morbidity. CPB, cardiopulmonary bypass; ScVO_2_, central venous oxygen saturation.

CPB duration was significantly longer, and the lowest temperature on CPB was significantly lower in patients with major morbidity or mortality.

Nadir ScVO_2 _during CPB was significantly lower, and peak lactate, significantly higher in patients with major morbidity and mortality.

**Figure 1:**
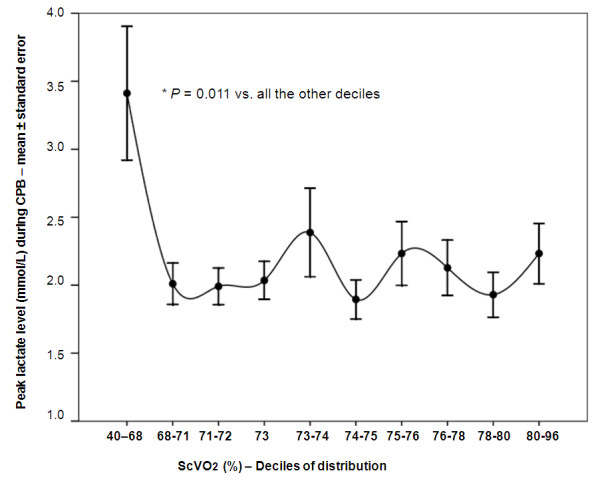
**Peak whole blood lactate according to the nadir ScVO_2 _value**. Significance assessed with the Kruskal-Wallis test.

At the nonparametric Spearman's correlation test, a trend (= 0.072) was noted toward a correlation between nadir ScVO_2 _and peak lactate. The better to explore this correlation, the patient population was divided into deciles of distribution, and for each decile, the mean value of peak lactates (± standard error of the mean) was calculated. The resulting analysis is graphically reported in Figure 1, with spline curve interpolation. In a Kruskal-Wallis analysis, the value of peak lactate did not significantly change for values of nadir ScVO_2 _above 68%. Conversely, patients in the first decile of distribution (nadir ScVO_2 _40% to 68%) had a significantly higher peak lactate value with respect to all the other deciles.

The association of nadir ScVO_2 _and peak lactate with major morbidity and mortality was explored by using a logistic regression analysis with odds ratios and 95% confidence intervals (Table 3). In a univariate analysis, both ScVO_2 _and peak lactate were significantly associated with major morbidity and mortality. When pooled together in a single logistic regression model, both the factors remained independently associated with major morbidity, but peak lactate remained the only independent factor for mortality.

**Table 3:** Crude and adjusted association (logistic regression analysis) between ScVO_2_, lactates, and major morbidity and mortality

Major morbidity					
**Analysis**	**Factor b**		**SEM**	***P *value**	**OR (95% CI)**

Crude	ScVO2	-0.136	0.03	0.001	0.87 (0.82-0.93)
	Constant	7.6	2.11		

Crude	Lactates	0.58	0.14	0.001	1.78 (1.35-2.36)
	Constant	-3.57	0.44		

Combined	ScVO2	-0.114	0.03	0.001	0.89 (0.84-0.95)
	Lactates	0.499	0.16	0.002	1.65 (1.2-2.26)
	Constant	4.87	2.28		

Adjusted	ScVO2	-0.117	0.03	0.001	0.89 (0.84-0.94)
	CPB time	0.01	0.003	0.002	1.01 (1.003-1.02)
	Constant	7.6	2.11		

Adjusted	Lactates	0.42	0.14	0.004	1.52 (1.15-2.03)
	CPB time	0.008	0.003	0.015	1.01 (1.003-1.01)
	Constant	-4.1	0.51		

**Mortality**					

**Analysis**	**Factor b**		**SEM**	***P* value**	**OR (95% CI)**

Crude	ScVO2	-0.114	0.03	0.001	0.89 (0.84-0.95)
	Constant	4.8	2.16		

Crude	Lactates	0.7	0.16	0.001	2 (1.46-2.76)
	Constant	-5.2	0.69		

Combined	ScVO2	-0.06	0.04	0.158	0.94 (0.87-1.02)
	Lactates	0.608	0.18	0.001	1.84 (1.3-2.61)
	Constant	-0.658	3.21		

Adjusted	ScVO2	-0.091	0.03	0.01	0.91 (0.85-0.98)
	CPB time	0.011	0.004	0.003	1.01 (1.004-1.02)
	Constant	1.8	2.5		

Adjusted	Lactates	0.56	0.17	0.001	1.75 (1.26-2.42)
	CPB time	0.009	0.004	0.032	1.01 (1.003-1.02)
	Constant	-5.97	0.87		

CI, confidence interval; CPB, cardiopulmonary bypass; OR, odds ratio; ScVO_2_, central venous oxygen saturation; SEM, standard error of the mean.

Other factors associated with major morbidity and mortality in a univariate logistic regression analysis were age, weight, Aristotle score, serum creatinine value, CPB duration, and lowest temperature on CPB. Because of the limited number of major morbidity and mortality events, and to avoid overfitting and multicollinearity of the model, only CPB duration was considered an adjustment factor. CPB duration is a single variable that indirectly represents the complexity of the operation and the need for low temperatures.

**Figure 2:**
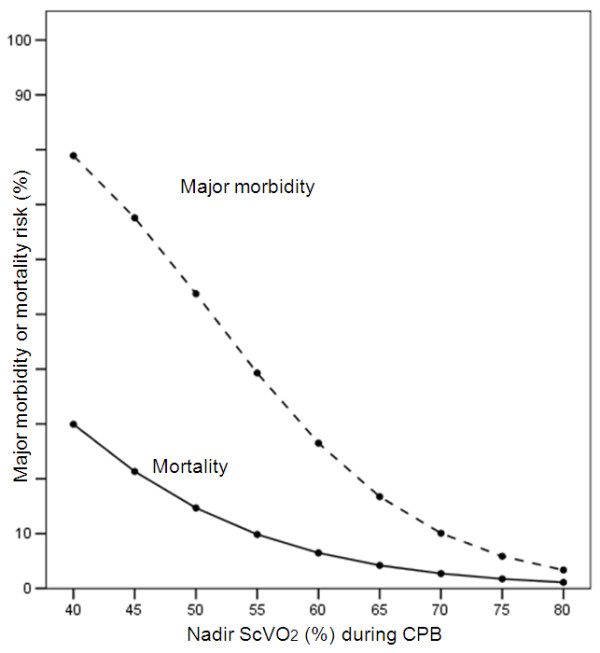
**Predicted major morbidity and mortality rates (logistic regression analysis) according to the nadir ScVO_2 _value, for a cardiopulmonary bypass (CPB) duration of 90 minutes**.

**Figure 3:**
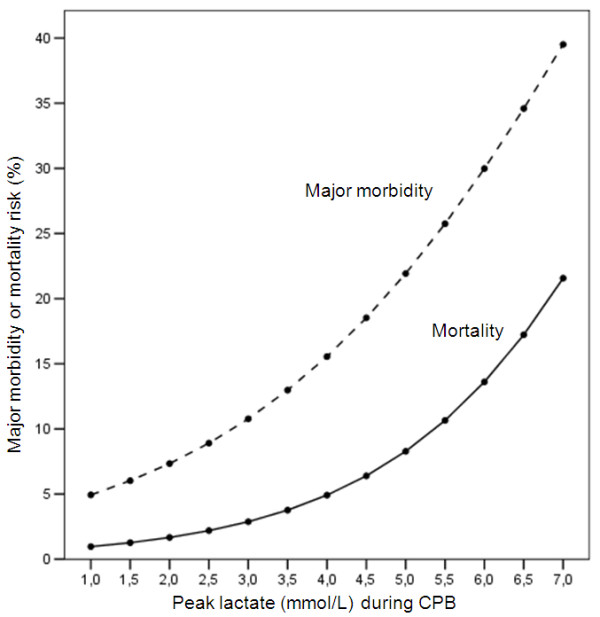
**Predicted major morbidity and mortality rates (logistic regression analysis) according to the peak whole blood lactate value, for a cardiopulmonary bypass (CPB) duration of 90 minutes**.

After adjustment for CPB duration, nadir ScVO_2 _and peak lactate remained significantly associated with major morbidity and mortality. This association is graphically presented in Figures 2 and 3, for a CPB duration settled at 90 minutes.

**Figure 4:**
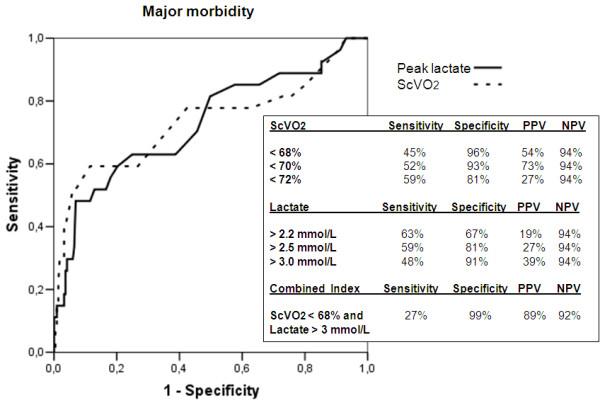
**Receiver operating characteristic curve for major morbidity**. Different cut-off values for nadir ScVO_2_, peak lactate, and a combined index are explored. PPV, positive predictive value; NPV, negative predictive value.

The ability of nadir ScVO_2 _and peak lactate to predict major morbidity and mortality was investigated by using an ROC analysis. For major morbidity (Figure 4), the AUC was comparable between the two predictors, being 0.73 (95% confidence intervals, 0.61 to 0.86) for nadir ScVO_2 _and 0.73 (95% confidence interval, 0.61 to 0.84) for peak lactate. Different cut-off points were explored for sensitivity, specificity, positive predictive value (PPV), and negative predictive value (NPV). Both the factors demonstrated a very high NPV (94%); the PPV of peak lactate was always low (< 40%), whereas a nadir ScVO_2 _value < 70% had a PPV of 73%.

**Figure 5:**
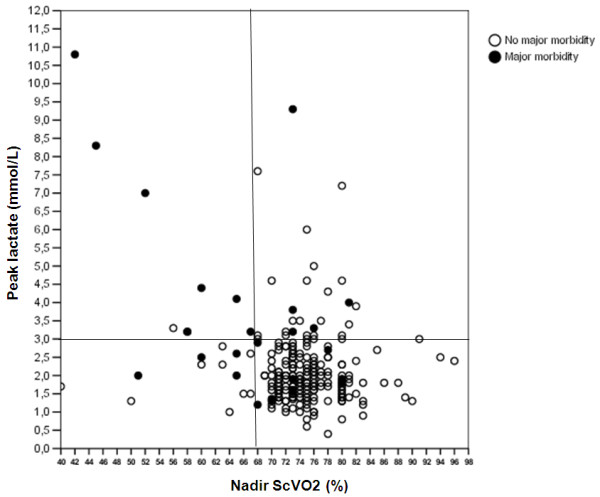
**Patient distribution according to the cut-off values of 68% (nadir ScVO_2_) and 3 mmol/L (peak lactate)**.

A combined index (nadir ScVO_2 _< 68% and peak lactate > 3 mmol/L) had the best PPV (89%) with a NPV of 92%. In Figure 5, the patient population is graphically analyzed with respect to this combined index. Nine patients are placed in the upper left quadrant (positive combined index), and eight had a major morbidity. Thirty-three patients had a peak lactate > 3 mmol/L, and in 30 cases, this value was observed during the rewarming phase. Twenty-two patients had a nadir ScVO_2 _< 68%, and in 20 cases, this value was observed during the rewarming phase.

**Figure 6:**
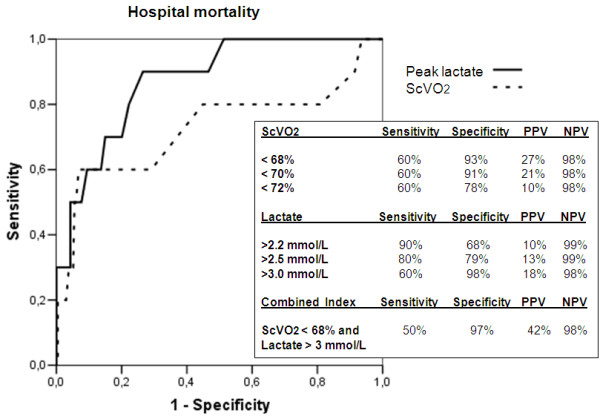
**Receiver operating characteristic curve for mortality**. Different cut-off values for nadir ScVO_2_, peak lactate, and a combined index are explored. PPV, positive predictive value; NPV, negative predictive value.

With respect to mortality (Figure 6), peak lactate had a higher accuracy than nadir ScVO_2_, with an AUC of 0.87 (95% confidence interval, 0.78 to 0.97) versus 0.73 (95% confidence interval, 0.52 to 0.94). Both the predictors had excellent NPV but a poor PPV at the various cut-off points explored. The combined index reached a PPV of 42%.

## Discussion

Low values of venous oxygen saturation during CPB are generally interpreted as an increased peripheral oxygen-extraction rate due to an oxygen delivery (DO_2_) inadequate to sustain the oxygen consumption (VO_2_). Under these conditions, the increased oxygen-extraction rate may satisfy the peripheral oxygen needs, until a certain value, without the need for anaerobic energy production. In adult patients during normothermic CPB, it was demonstrated that this mechanism may cover the oxygen needs unless the DO_2 _falls below a critical value, settled at around 260 mL/min/m [8]. Below this value, a progressive increase of blood lactate is found, as a marker of anaerobic energy production.

In our series of 256 pediatric patients, this pattern was confirmed for ScVO_2 _values below 68%, with a significant increase of peak lactate during CPB, and may be interpreted as a condition of increased oxygen-extraction rate, insufficient to cover the VO_2_, with activation of anaerobic energy production (upper left quadrant of Figure 5).

ScVO_2 _values below the normal range (lower left quadrant of Figure 5) may be interpreted as a condition of increased oxygen-extraction rate, sufficient for covering the VO_2_. It may be hypothesized to be even a time-related factor, so that these patients may be in an early phase of dysoxia, and that this phase did not last enough to bring them into the anaerobic energy-production zone.

Hyperlactatemia is a complex condition that may result from several mechanisms. Type A hyperlactatemia is defined as an impaired tissue oxygenation, leading to increased anaerobic metabolism and an excessive production of pyruvate (which is then converted to lactate), and numerous studies have established the use of lactates as a marker of global tissue hypoxia in circulatory shock.

Type B hyperlactatemia is dependent on a number of factors not directly related to a tissue dysoxia, basically representing the inability of the peripheral tissues to use oxygen. Lactate concentration depends on the balance between production and elimination (by the liver). However, the kinetics of lactates clearance depends basically on the production rate, because hepatic clearance appears to be preserved even during cardiogenic shock [18]. Nonetheless, in conditions of severe splanchnic hypoperfusion, the hepatic blood flow declines, the liver capacity to use lactates is decreased, and the liver itself may become a producer of lactate [18].

Apart from these two basic mechanisms leading to hyperlactatemia, a hypothesis suggests that lactate production is not always linked with anaerobic metabolism, rather representing a fuel source used during stress conditions [19]. This hypothesis is, however, primarily based on exercise-induced hyperlactatemia.

Whereas hyperlactatemia coupled with low ScVO_2 _may be easily ascribed to type A, hyperlactatemia with normal ScVO_2 _values (upper right quadrant of Figure 5) is more difficult to interpret. This condition is not rare in our series (24 patients, 9.4% of the total), but is associated with major morbidity in only 20% of the cases, whereas hyperlactatemia with low ScVO_2 _values leads to major morbidity in 89% of the patients. Our interpretation is that this patient population may have experienced a "reperfusion phenomenon" during the rewarming phase, with peripheral districts previously excluded from the circulation by a hypothermic vasoconstrictive reaction.

The analysis of our data supports the concept that both ScVO_2 _and lactate should be considered during CPB, and that the most relevant information is provided by a combined index (ScVO_2 _< 68% + lactate > 3 mmol/L), which yields a relevant PPV of 89% in predicting postoperative major morbidity and an acceptable 42% for mortality.

From the clinical point of view, the relevant information is more related to the NPV than to the PPV of both the indices. Actually, our data demonstrate that patients who did not experience low values of ScVO_2 _and/or high values of peak lactate had an outcome free from adverse events in the great majority of the cases.

The condition of type A hyperlactatemia was detected in the majority of the cases during the rewarming phase. It is likely that, because of the increased oxygen demands, this phase is at higher risk for organ dysoxia. Our data are in agreement with Munoz and associates [15], who demonstrated that peak lactate developed mainly during the rewarming phase, and that the increase of lactate during CPB was associated with increased morbidity and mortality in congenital heart disease operations. However, these authors recognized that, despite good sensitivity and specificity, the PPV of blood lactate-derived indices was poor for mortality (23%) and acceptable for morbidity (45%). Similar values were found in our series for isolated blood lactate indices (18% for mortality and 39% for major morbidity).

Even recognizing the important value of blood lactate during CPB, these measurements have two limitations: (a) noncontinuous measurement, and (b) time-related changes. This second limitations is due to the fact that once formed, lactate takes time to be cleared off, and this time depends on a number of factors, including the existence of an ongoing dysoxia and the liver ability to clear lactates (in turn dependent on liver perfusion).

Conversely, ScVO_2 _may be continuously measured (with our or other techniques, including surgical positioning of oximetry catheters or oximetric cells placed inside the venous line of the CPB circuit), and rapidly recovers normal values once the DO_2 _returns to be adequately matched with the VO_2_.

The option of using an oximetric CVC may, however, offer many advantages. The CVC is inserted during the monitoring maneuvers, before the surgery onset; it may therefore provide useful information during the surgical phases before going on CPB. Moreover, it offers ScVO_2 _values after CPB discontinuation and during the ICU stay. This information has been proven as very relevant in high-complexity operations for congenital heart defects [5,6].

In recent years, near-infrared spectroscopy (NIRS) has been proposed as a surrogate of central or mixed venous oxygen saturation in the setting of pediatric cardiac surgery. The main advantages of NIRS are the continuous monitoring during and after the operation, and the noninvasiveness. The NIRS-derived regional oxygen saturation (rSO_2_) may be measured at a cerebral level or even at a somatic level, with electrodes placed on the frontal skull or the abdominal wall. Preoperative low (< 50%) values of rSO_2 _have been associated with an increased mortality in children undergoing congenital heart surgery [20]. rSO_2 _is different from ScVO_2_, central SVO_2_, or jugular bulb SVO_2_. However, many studies demonstrated that rSO_2 _is correlated with the other venous oxygen saturation measurements, usually providing lower values, but being consistent in relative changes over time [21-25]. Recently, we demonstrated that continuously measured ScVO_2 _correlates with NIRS before, during, and after CPB in pediatric patients undergoing cardiac operations [26]. Only a limited number of patients in our series received NIRS monitoring, and we cannot therefore explore the role of rSO_2 _as a predictor of adverse outcome. However, in the setting of adult cardiac surgery, rSO_2 _has been used with good results for goal-directed therapy, and low values of rSO_2 _have been associated with adverse outcomes [27].

Some limitations of our study exist. First, the retrospective nature, with a selection bias toward operations of moderate to severe complexity. Second, the limited number of events in our series does not allow us to account for the role of all the possible confounders with a complete multivariable analysis. Third, the patient population includes neonates, infants, and children, and this may be a source of bias. Finally, continuous ScVO_2 _measurement during CPB may be limited by a number of factors already mentioned in our previous studies [17,25]. Positioning problems and interference with the surgical field light may limit the applicability of this technique during CPB. ScVO_2 _measurement, once the superior vena cava is cannulated and tightened, offers information that is limited to the upper part of the body, with a major contribution from the brain circulation. This may be useful for a more selective monitoring of the adequacy of brain perfusion, but leaves unexplored the adequacy of visceral perfusion during CPB.

## Conclusions

Our study supports the use of continuous monitoring of venous oxygen saturation during CPB in congenital heart operations, with blood lactate measurement that should be serially repeated whenever the ScVO_2 _decreases below a value of 68%. Detection of a blood lactate value higher than 3 mmol/L under these conditions should be considered a warning signal for inadequate DO_2_.

Of course, the observation that low values of ScVO_2 _and high values of peak lactates are associated with bad outcomes does not allow us to conclude that goal-directed strategies aimed to increase the DO_2 _during CPB may be beneficial in pediatric cardiac surgery.

Our observation only generates the hypothesis that whenever the ScVO_2 _is < 68% with concomitant hyperlactatemia, efforts should be applied to increase the DO_2_. This may include increasing the pump flow, using systemic vasodilators, modulating cerebral blood flow with an adequate arterial pCO_2 _management, and increasing the hemoglobin value through hemofiltration and/or packed red cells transfusions. This goal-directed strategy offered significant advantages in the setting of adult cardiac surgery (26), but only a prospective randomized study may demonstrate the same beneficial effects in the pediatric patients undergoing cardiac surgery. Further studies in this area should also consider the "time-related factors," like the duration of a low ScVO_2 _condition and the kinetics of lactate formation.

## Key messages

• In a population of pediatric (younger than 6 years) patients undergoing cardiac operations with CPB, the lowest value (nadir) of ScVO_2 _during CPB was predictive for postoperative major morbidity and mortality.

• Patients who experienced a nadir ScVO_2 _value < 68% during CPB developed hyperlactatemia (> 3 mmol/L) during CPB.

• Hyperlactatemia during CPB was associated with an increase in the postoperative major morbidity and mortality rate.

• The best combination of positive and negative predictive values for major postoperative morbidity was obtained for a combined index (ScVO_2 _< 68% and blood lactate > 3 mmol/L).

## Abbreviations

AUC: area under the curve; CPB: cardiopulmonary bypass; CVC: central venous catheter; DO_2_: oxygen delivery; ICU: intensive care unit; NIRS: near-infrared spectroscopy; NPV: negative predictive value; PPV: positive predictive value; RBC: red blood cell; ROC: receiver operating characteristic; rSO_2_: regional oxygen saturation; ScVO_2_: central venous oxygen saturation; VO_2_: oxygen consumption.

## Competing interests

The authors declare that they have no competing interests.

## Authors' contributions

MR contributed to study design, statistical analysis, and manuscript preparation. CC participated in data acquisition and interpretation. GI provided data acquisition and interpretation and manuscript drafting. TDT and SE were involved in data acquisition. AF contributed to data interpretation and manuscript drafting.
